# Positive irrational beliefs are associated with hypomanic personality

**DOI:** 10.3389/fpsyg.2023.1053486

**Published:** 2023-03-20

**Authors:** Alexandru I. Tiba, Simona Trip, Carmen H. Bora, Marius Drugas, Feliciana Borz, Daiana C. Miclăuş, Laura Voss, Sorin C. Iova, Simona Pop

**Affiliations:** ^1^Department of Psychology, University of Oradea, Oradea, Romania; ^2^Hull York Medical School, Hull, United Kingdom; ^3^Faculty of Medicine and Pharmacy, University of Oradea, Oradea, Romania

**Keywords:** irrational beliefs, depression, hypomania, REBT, risk, mania and bipolar disorder

## Abstract

Primary irrational beliefs, such as demanding about attaining personal goals, are a common trans-diagnostic factor involved in many emotional disorders. Although Bipolar Disorder (BPD) is a severe emotional disorder, little is known about the role of primary irrational beliefs in the risk of BPD. Given that the risk for mania is related to responses to positive rather than adverse events, we developed a measure of irrational beliefs in response to cues of positive events. This is the first study that examines the relationship between positive primary irrational beliefs and the risk of BPD. 119 participants completed an online survey including measures for the risk of BPD, irrational beliefs, positive irrational beliefs, mania-related cognitions, and mood measures (depressive and manic mood). Results revealed significant associations between the risk of BPD and positive primary irrational beliefs, irrational beliefs, positive generalization, and mood. Regression analyses revealed that positive primary irrational beliefs, such as demanding to attain significant goals in response to cues for positive events, uniquely predict the risk for BPD independently of mood, mania-related cognitions and irrational beliefs. These findings encourage the treatment approaches focused on restructuring primary irrational beliefs in response to positive situations to reduce the risk of BPD.

## Introduction

Bipolar disorder (BPD) is a highly severe mental disorder (Vigo et al., 2016). Added to its severity, the poor treatment adherence and the limited effectiveness of existing interventions (Ye et al., 2016) complicate its treatment. Several meta-analyses showed that cognitive-behavioral therapies (CBT) are an effective adjunctive treatment for BPD, reducing the relapse rate, depressive symptoms, mania severity, and resulting in an increased psychosocial functioning (Szentagotai and David, 2010; Ye et al., 2016; Chiang et al., 2017; Miklowitz et al., 2021). Yet, some effects are short-lived and controversial (Ye et al., 2016). The need to improve the existing treatments and preventive interventions for BPD has oriented the research efforts on elucidating the psychological mechanisms involved in the risk of developing the disorder. Finding new mechanisms amenable to change by psychotherapy interventions, is a very promising avenue (Szentagotai and David, 2010). Cognitive factors are such examples of treatment targets that can offer a great promise for improving the psychological treatment of BPD. However, research on cognitive processes underlying the risk for BPD yielded mixed results about the types of cognitions involved in the risk of BPD (Yesilyaprak et al., 2019). Recent research has consistently pointed toward dysregulation of goal representations and approach behavioral systems as significant risk factors for BPD (Johnson, 2005; Johnson et al., 2010). Unfortunately, these findings have not consistently informed the existing treatments. A possible answer may come from looking into existing trans-diagnostic cognitive mechanisms involved in emotional disorders for which effective treatment methods have already been tested. Responding with primary irrational beliefs (e.g., absolute demands for attaining personal goals or desires: “*I must have what I want*”) (Ellis, 1994; Ellis et al., 2010; Dryden, 2019) when adverse events block someone’s goals is one form of goal dysregulation that has been consistently shown as a mechanism for various emotional disorders and dysfunctions (Tiba et al., 2012; Vîslã et al., 2016; David et al., 2019).

Furthermore, there is substantial evidence for the effectiveness of psychotherapy methods (e.g., Rational Emotive Behavior Therapy) in changing irrational beliefs (David et al., 2018, 2019). Lately, both empirical and theoretical studies have linked rigid goals in form of demandingness to dysregulated behavioral activation system (BAS) (Tiba, 2005; Yıldırım and Maltby, 2021), increased activation after goal-attainment (Tiba and Szentagotai, 2005), and exaggerated active coping in response to both adversities (Tiba, 2010; Tiba and Manea, 2018a,b). Being a well-known trans-diagnostic mechanism for emotional disorders (Vîslã et al., 2016) for which we have effective treatment methods (Trip et al., 2010; David et al., 2018, 2019), evidence of the role of primary irrational beliefs in vulnerability to BPD may provide a strong treatment target, spurring the development of better psychological interventions for the treatment or the prevention of BPD.

Unfortunately, to our knowledge, no study has investigated irrational primary beliefs or rigid beliefs in relation to the risk of BPD and mania. One reason for this lack of research is that primary irrational beliefs were measured as responses to negative situations (Macavei and McMahon, 2010). Primary irrational beliefs or rigid beliefs are usually defined referring to the situation in which one person demands that he should meet personal goals and desires when adverse events interfere with his goal attainment (Ellis, 1994). Instead, the risk for manic episodes is related to responses to positive events and not to negative events (Johnson et al., 2000; Gruber et al., 2008). Moreover, dysfunctional responses involved in the risk for mania are mainly related to specific types of positive events, such as goal-attainment related positive events (Johnson et al., 2000). Thus, the types of positive cognition involved in the risk for mania are expected to follow the situation of attaining personal goals. Given the role of positive events as activating events for the risk for mania, the existing measurements of irrational beliefs may not be appropriate to test their involvement in the risk for BPD and the development of episodes of mania.

In the present study, we overcome these shortcomings by measuring irrational beliefs in positive situations. We examined whether primary irrational beliefs (Ellis et al., 2010) about attaining goals in positive situations predict hypomanic personality which is a well-known risk factor of bipolar disorder beyond other cognitive and affective factors such as hypo/manic states, depressive mood, and positive generalizations.

### The present study

In the present study, we investigated whether irrational beliefs in positive situations were uniquely related to the risk of hypo/mania measured by the Hypomanic Personality Scale (HPS). We asked participants to measures related to the risk for hypo/mania, HPS and a measure of positive irrational beliefs. Then, we used regression analyses to examine whether positive irrational beliefs have an unique contribution to the hypo/mania risk (measured by HPS) controlling for the contribution of demographic variables (age and sex), depressed and manic mood, and positive hypo/manic thoughts. To develop a measure of positive irrational beliefs, we followed the suggestion of Leahy and Beck (1988), according to which cognitive schemas in mania are the mirror image of cognitive schemas in depression (Leahy and Beck, 1988). Based on this suggestion, we developed positive items mirroring items based on a well-known measure of irrational beliefs. We used comfort, achievement and approval-related items from the General Attitude and Beliefs Scale (GABS scale; DiGiuseppe et al., 1988; Bernard, 1998) and developed by positive correspondence a positive irrational measure. We mirrored four types of irrational appraisals: demandingness (e.g., “It’s essential to be liked by important people, and I will not accept their not liking me”) into positive demandingness (e.g., “When I see important persons like me, I consider being liked by them as a necessity because it is essential to be liked by important persons”), low frustration tolerance (e.g., “I can’t stand not doing well at tasks that are important to me”) into “positive overwhelming” (e.g., “I am overwhelmed by happiness when I do well tasks that are important for me”), awfulizing (e.g., “It’s awful to have hassles in one’s life and it is a catastrophe to be hassled”) in wonderfulizing (e.g., “It is wonderful to have daily uplifts in your life and it is extraordinary when this happens”) and self-depreciation (e.g., “If I do not perform well at tasks that are so important to me, it is because I am a worthless bad person”) into self-appreciation (e.g., “When I succeed in something important I am a good and valuable person”). Each process referred to three content types: achievement, comfort, and social approval. The items we developed by the first author (A.T.). Two psychologists (A.T. and C.B.) have evaluated the mirror correspondence of the items. We analyzed the relationship between positive primary irrational beliefs and hypo/mania risk (HPS) along with other mania and cognitive related measures in the general population.

## Materials and methods

### Participants

We recruited an online sample of 119 participants for this study. Participants were recruited using social media adverts for undergraduates enrolled at the university and contacting undergraduate students who took part in previous research at university. No incentives to participate were provided. Two participants did not give their consent for participation and were excluded. Additionally, nine subjects who failed at attention check questions were eliminated. One participant was eliminated being below 18 years of age at the enrolment in the study. The final sample consisted of 107 participants (Mean*_*age*_* = 25.11 years, SD = 10.00; 83 females, 24 males) (see Table 1).

**TABLE 1:** Sample characteristics and the main variables.

Sample characteristics	Descriptive statistics
Age in years, mean (SD, range)	25.11 (± 10.00.18–63)
**Gender identity, *n/N* (%)**	
Female	83/107 (77.6%)
Male	24/107 (22.4%)
HPS, mean (SD, range, *n*)	19.60 (± 8.62, 3–38, *n* = 107)
**Irrationality in adverse situations scores (HABS) (SD, range, *n*)**	
Irrationality subscale	34.43 (± 9.71, 12–56, *n* = 107)
Achievement related irrationality	16.14 (± 3.93, 5–24, *n* = 107)
Demandingness	10.64 (± 2.63, 3–15, *n* = 107)
**Positive irrationality scores (SD, range, *n*)**	
Positive demandingness	14.05 (± 3.23, 4–20, *n* = 107)
Sociotropic positive irrational beliefs	33.42 (± 9.98, 10–50, *n* = 107)
Autonomy-related positive irrational beliefs	101.72 (± 18.09, 52–130, *n* = 107)
General positive irrational beliefs	138.61 (± 25.14, 74–185, *n* = 107)
POG, mean (SD, range, *n*)	52.57 (± 12.36, 16–78, *n* = 107)
PHQ-2, mean (SD, range, *n*)	2.67 (± 1.45, 0–6, *n* = 107)
ASRM, mean (SD, range, *n*)	5.13 (± 3.62, 0–15, *n* = 107)

HPS, Hypomanic Personality Scale; PHQ-2, patient health questionnaire 2; ASRM, altman Self-Rating Scale for Mania; POG, Positive Generalization Scale.

### Measures

#### Depression

Participants completed the Patient Health Questionnaire 2 (PHQ-2; Kroenke et al., 2003). The PHQ-2 is a 2-item self-report measure developed to screen for symptoms of depression. Participants had to endorse how much they had “little interest or pleasure in doing things” (Kroenke et al., 2003) and were “feeling down, depressed or hopeless” (Kroenke et al., 2003) over the previous 2 weeks on a 4 points Likert scale from 0 (not at all) to 3 (nearly every day) (Cronbach α was 0.63).

#### Irrational beliefs in negative situations

To measure irrational beliefs, we used a variant of the ABS2-short form scale focused on achievement (HABS2-short form; Hyland et al., 2014). HABS2-short form contains 24 items extracted from a 72 items widely used irrational beliefs measure (ABS-2) reflecting four irrational cognitive processes (demandingness, low-frustration tolerance, awfulizing, and self-depreciation) mainly about achievement and comfort (DiGiuseppe et al., 2020). We summed the responses from rational items to calculate the rational subscale score Cronbach α was 0.89) and the responses from irrational items to calculate the irrational subscale score (Cronbach α was 0.80). Given the relevance of the content-specific irrational beliefs for different pathologies (DiGiuseppe et al., 2021), and the suggestion that individuals with manic pathology have dysfunctional responses to achievement related-events (Johnson et al., 2000), we additionally calculated two scores: an irrational achievement score (Cronbach α was 0.74) and a rational achievement scale each containing five items (Cronbach α was 0.85).

#### Hypomanic personality

The Hypomanic Personality Scale (HPS; *Eckblad and Chapman, 1986*) is a self-report scale that measures the risk of bipolar disorder. Participants had to endorse 48 items using a true-false response format. Items refer to emotional, *behavioral* and cognitive tendencies that *characterize* hypomanic personality (e.g., “I am frequently in such high spirits that I can’t concentrate on any one thing for too long.”; “I often feel excited and happy for no apparent reason” (Cronbach α was good 0.87).

#### Hypo/mania symptoms

The Altman Self-Rating Scale for Mania (ASRM; Altman et al., 1997) measure the experience of hypo/manic symptoms over the past week. Participants rate their responses on a Likert scale from 1 (e.g., “I do not feel happier or more cheerful than usual”) to 5 (“I feel happier of more cheerful than usual all the time”). The scores were recoded from 0 to 4 to compare the responses to the recommended cut-off of 6 (Altman et al., 1997) (Cronbach α was acceptable 0.74).

#### Positive hypo/manic thoughts

The Positive *Generalization* Scale (POG; *Eisner et al., 2008*) contains 16 items that measure the over-*generalization* people make from successes (e.g., “When something good happens to me, it makes me expect good things in other parts of my life too”). Participants had to indicate how much they disagree with each item on a Likert type scale from 1 (I disagree a lot) to 4 (I agree a lot) (Cronbach α was good 0.89).

#### Positive irrational beliefs measure

Thirty-seven positive irrational statements mirroring to four types of irrational appraisals from the General Attitude and Beliefs scale (GABS scale; DiGiuseppe et al., 1988; Bernard, 1998) were included in the analyses: positive demandingness (e.g., *“When I see I am about to know I will succeed, for me succeeding becomes a must not just a desire*”), positive “overwhelming” (e.g., “*It is the greatest happiness to succeed in what is important and I’m overwhelmed by my happiness when I do*”), “wonderfulizing” (e.g., *“When I perform well in something important, it is wonderful”*), and self-valuing (e.g., “*When I succeed in something important I am a good and valuable person”*). Thus, we mirrored thirty-four items about comfort, achievement, approval and self-worth from GABS scale. Since only one item of demandingness items was negatively formulated, we included the original items and added three new items reflecting demandingness in response to positive situations (see the positive primary irrational statements below). Participants rated how much they agreed with each statement on a Likert type scale from 1 (totally disagree) to 5 (totally agree). The scale was previously tested in a pilot study of fifteen participants, results showing that participants understood the items. Based on participants feedback the items were adjusted to better reflect their experiences in response to positive events. Principal axis factoring with Promax rotation was run to establish the factorial structure of the positive irrational belief items. The Kaiser-Meyer-Olkin test value was 0.915 suggesting that the sample size in this study was sufficient for exploratory factorial analysis. The Bartlett’s Test of Sphericity χ2 = 3394.657, df = 666 was significant, *p* < 0.001 showing that the matrix of relationships between the items differs from the unit matrix without relations. The scree plot inspection and the Monte Carlo parallel analysis offered a two factor solution: (1) a sociotropy positive irrational beliefs factor (10 items, Cronbach α 0.94) and (2) an autonomy positive beliefs factor (26 items, Cronbach α 0.96). We removed the item eight because it did not load into either of the two factors. We calculated two additional scores: (1) a general score for all the items (the internal consistency coefficient Cronbach α was excellent 0.96), and (2) a positive primary irrational beliefs score (i.e., “When I see I am about to know I will succeed, for me succeeding becomes a must not just a desire”; “When I realize that someone important for me finally appreciates me, being liked and appreciated by that person becomes a necessity for me”; “When I realize that my life finally becomes pleasurable and comfortable, having a pleasant life becomes a necessity for me”; “When I see important persons like me, I consider being liked by them as a necessity because it is essential to be liked by important persons”(Cronbach α was 0.67).

### Procedure

Data collection was conducted online from March 15th to June 9th 2022. Participants were recruited by adverts on social media university groups and by sending an online link with an invitation for study. After accessing the link, participants read the study description and the informed consent form. Participants who gave their consent, responded to demographic questions and completed the study measures. HPS, PHQ-2, HABS short form, Altman scale, POG, and the positive irrational beliefs measures were included in the present study. Additional measures were completed, such as Positive events scale, Profile of affective distress (Opris and Macavei, 2007), additional irrational beliefs items, and an emotional regulation rating form all which were subjected to different analyses for a second study. To keep a minimal number of items, PHQ-2 scale was selected for this study. The study received ethical approval from the ethical committee of the Faculty of Socio-Humanistic Studies, University of Oradea (approval no 516/15.03.2022).

### Data analysis plan

Data analysis plan first involved data screening and preliminary analyses. We used parametric correlational analyses for analysis of the associations between the variables. Linear regression model was used to analyze the prediction of HPS by positive primary irrational beliefs. Principal axis factoring with Promax rotation was used to analyze the structure of thirty-seven positive irrational beliefs items. The factorial, correlational and regression analyses were carried out using IBM SPSS Statistics for Windows, Version 23.0, IBM Corp., Armonk, NY, USA. Multicollinearity analyses showed that all values of the variance inflation factor (VIF) were below the maximum threshold level of 10 (all VIFs < 2) and all tolerance levels were above the value of 0.5. The visual inspection of the P-P plot and scatterplot suggests a normal distribution of residuals and that the data meet the homoscedasticity assumption. Exploratory analyses investigated moderation roles of positive overgeneralizations and the current mood in the relationships between positive irrational beliefs and bipolar vulnerability. The calculated power based on seven predictors yielded an acceptable power (0.826) using a 107 sample and a medium effect size (Statistics Kingdom, 2017).

## Results

### Descriptive statistics

The means, standard deviation, minimum, and maximum scores for the main study variables (age, depression level, hypo/mania, bipolar vulnerability, irrational beliefs, and positive generalizations) are described in Table 1. Analyses of skewness and kurtosis suggest data met the assumptions of normality (all coefficients were below the cut-off point <1). The Kolmogorov–Smirnov test normality suggest normal distributions (all *p*s > 0.01) for hypomanic personality, total positive irrational beliefs score, irrational beliefs, sociotropic positive irrational belief, autonomy-related positive irrational beliefs, and positive overgeneralizations but not for positive primary irrational beliefs, Altman Scale, and PHQ-2 scale (all *p*s > 0.01). Regarding the level of depression, 50.5% (*N* = 54) of the sample scored above the PHQ-2 cut-off score of 3 or more, suggesting clinical depression (Kroenke et al., 2003). Regarding hypo/manic symptoms, 44.9% (*N* = 48) of the sample scored above the ASRM cut-off of 6, suggesting the possibility of hypo/mania (Altman et al., 1997).

### Correlation analyses

Spearman correlation coefficients Rho between the non-parametrically distributed variables (Altman scale, positive primary irrational beliefs, PHQ-2) and Pearson coefficients for normally distributed variables (HPS, positive generalizations, total positive irrational beliefs, autonomy-related positive irrational beliefs, sociotropic positive irrational beliefs, and total positive irrational beliefs) are described in Table 2. According to our hypotheses, the Hypomanic Personality Scale scores were positively correlated with the level of depression and hypomania, positive generalizations (marginally *p* = 0.056), irrational beliefs, and with primary irrational beliefs measured in positive situations. Positive primary irrational beliefs or rigid pursuit of goals in positive situations (positive demandingness) (and all types of positive irrational beliefs) were positively associated with scores on the Hypomanic Personality Scale, but not with state measures of hypo/mania and positive generalizations. Irrational beliefs were positively associated with the risk of hypo/mania, depression, and all types of positive irrational beliefs and negatively related to positive generalizations. Although the risk of hypo/mania was significantly related to general irrational beliefs (*p* = 0.042), we observed no significant relationships between the risk of mania and achievement-related irrational beliefs (*p* > 0.05). Furthermore, partial correlation analyses showed that controlling for positive primary irrational beliefs yields the relation between irrational beliefs and the risk of hypo/mania non-significant (*r* = 0.027, *p* = 0.787).

**TABLE 2:** Correlation coefficients for the study variables.

Variables	1	2	3	4	5	6	7	8
1. HPS	–							
2. PHQ-2	0.222*	–						
3. ASMR	0.412**	0.032	–					
4. POG	-0.185	0.271**	-0.224**	–				
5. IBs	0.197*	0.566**	-0.036	-0.283**	–			
6. PPIBS	0.357**	0.145	0.020	0.011	0.476**	–		
7. APIBs	0.327**	0.116	0.096	-0.006	0.251**	0.686**	–	
8. SPIBs	0.315**	0.104	0.099	-0.010	0.500**	0.849**	0.479**	–
7. TPIBs	0.373**	0.130	0.117	0.007	0.388**	0.856**	0.933**	0.761**

PHQ-2, patient health questionnaire 2; ASRM, Altman Self-Rating Scale for Mania; POG, Positive Generalization Scale; IBs, irrational beliefs; APIBs, autonomy-related positive irrational beliefs subscale; SPIBS, sociotropic positive irrational beliefs; PPIBs, positive primary irrational beliefs; TPIBs, total score for positive irrational beliefs; HPS, Hypomanic Personality Scale; *p < 0.05, **p < 0.01.

### Regression analyses

A hierarchical linear regression analysis was used to test the unique contribution of positive primary irrational beliefs to the Hypomanic Personality Scale whilst controlling for participant age, sex, current mood (PHQ-2 and ASRM), positive overgeneralizations, and irrational beliefs. The risk of hypo/mania was significantly related to the general measure of irrational beliefs in negative situations and not to achievement related irrational beliefs. Thus, we used the general irrational beliefs score in the future analyses.

The overall regression model was significant, *F*(7,98) = 11.686, *p* < 0.001, and explained 45% of variance in HPS scores. Table 3 details the regression coefficients between the study variables with HPS scores as the outcome.

**TABLE 3:** Regression analyses of positive primary irrational beliefs predicting hypomanic personality.

Step	Predictor	B (unstandardized)	S. E.	B (standardized)	*t*	C. I. (95%)
1	Sex	4.227	1.846	0.205	2.290**	0.567–7.887
	Age	-0.391	0.077	-0.455	-5.073**	−0.544 to −0.238
2	Sex	3.517	1.671	0.171	2.104*	0.200–6.833
	Age	-0.353	0.074	-0.410	-4.746**	−0.500 to −0.205
	PHQ-2	-0.150	0.602	-0.025	-0.250	−1.345 to 1.044
	ASMR	0.718	0.196	0.302	3.672**	0.330–1.107
	POG	-0.151	0.059	-0.216	-2.545*	-0.269 to −0.033
	IBs	0.220	0.086	0.248	2.565*	0.050–0.391
3	Sex	2.981	1.604	0.145	1.858	−0.202 to 6.165
	Age	-0.337	0.071	-0.392	-4.737**	−0.478 to −0.196
	PHQ-2	0.199	0.585	0.034	0.340	−0.961 to 1.359
	ASMR	0.713	0.187	0.300	3.815**	0.342–1.083
	POG	-0.125	0.057	-0.180	-2.193*	−0.239 to −0.012
	IBs	0.055	0.096	0.062	0.573	−0.136 to 0.247
	PPIBs	0.772	0.237	0.289	3.254**	0.301–1.242

PHQ-2, patient health questionnaire 2; ASRM, Altman Self-Rating Scale for Mania; PE, positive emotion subscale; POG, Positive Generalization Scale; IBs, irrationality in negative situations; PPIBs, positive primary irrational beliefs; HPS, Hypomanic Personality Scale; *p < 0.05, **p < 0.01.

In step 1, age and sex variables were entered. In step 2, PHQ-2, ASRM, positive generalizations, and irrationality in negative situations (HABS scale) were added. In step 3, the positive primary irrational beliefs were entered. Adding the ratings for the current mood, positive generation and irrational beliefs in step 2 significantly improved the fit of the model (Δ*R*2 = 0.187, Δ*F*(4, 99) = 7.651, *p* < 0.001), age, current hypomanic mood and positive generalizations significantly predicting the risk for hypomania. Adding positive primary irrational beliefs in step 3 did also significantly improves the fit of the model (Δ*R*2 = 0.059, Δ*F*(1, 98) = 10.589, *p* = 0.002). In the final model, ratings for positive primary irrational beliefs significantly predicted higher the risk for hypomania (β = 0.772, *p* = 0.002) beyond the current hypomanic mood (β = 0.713, *p* < 0.001), positive generalizations (β = −0.125, *p* = 0.031), and irrational beliefs about adverse events (*p* > 0.05).

As shown in Table 3, positive primary irrational beliefs positively predict HPS scores after controlling for age, participant sex, and current mood symptoms (PHQ-2 and ASRM), positive overgeneralizations and irrational beliefs in response to achievement related negative situations. Positive primary irrational beliefs were a significant positive predictor of HPS scores, showing that increased vulnerability to bipolar disorder is characterized by holding rigid beliefs about the attainment of goals and desires to cues of reaching personal goals.

### Ancillary analyses

To check the robustness of the findings, we run the same regression analyses using different measures of positive irrational belief. Results showed that all positive irrational beliefs, such as the global score of positive irrational beliefs (β = 0.083, *p* = 0.005), sociotropic positive irrational beliefs (β = 0.178, *p* = 0.030), and autonomy-related positive irrational beliefs (β = 0.097, *p* = 0.013) predict HPS beyond demographic and mania related variables. Furthermore, we explored potential moderators using the Model 1 of Hayes (2013) PROCESS for SPSS. Thus, we analyzed the moderation of the relationship between positive irrational beliefs and hypomanic personality by potential moderators: positive overgeneralizations and irrational beliefs in negative situations. Similar to previous studies investigating the relationship between dysfunctional dimensions of goals and the hypomanic personality (Dempsey et al., 2020), current mood (hypomanic and depression level) was included as covariate in these analyses. We observed no significant interaction effects between positive overgeneralization (interaction effect *p* = 0.908) or irrational beliefs in negative situations (interaction effect *p* = 0.497) and positive primary irrational beliefs in predicting vulnerability for bipolar disorder.

## Discussion

This study has investigated whether hypomanic personality, which is a risk factor for bipolar disorder, is associated with primary irrational beliefs (rigid pursuit of goals) in positive situations. Our results demonstrate that the risk of bipolar disorder (hypomanic personality) is associated with irrational beliefs, a well-known trans-diagnostic factor involved in vulnerability for emotional disorders. Especially, we showed that individuals who are more inclined to demand rather than just desire to attain their important goals when cues signal the possibility of goal attainment have higher levels of risk for developing hypo/mania. Although individuals who respond with high levels of irrational beliefs to adverse events have also higher risk of hypo/mania, our results suggest that their increased risk for mania depends on whether they keep responding using irrational demands to positive situations. Current depressed, hypomanic mood, and positive generalizations also characterized vulnerability for bipolar disorder. We found that positive primary irrational beliefs have a unique contribution to bipolar vulnerability beyond mood, positive generalizations, and irrational beliefs related to adversities. To our knowledge, this is the first study to prove that hypomanic personality which is a well-known vulnerability to bipolar disorder is linked to positive primary irrational beliefs. Our results are in line with studies showing a significant goal dysregulation in individuals at risk of bipolar disorder (Johnson et al., 2005; Johnson and Carver, 2006). Previous research showed that individuals with bipolar disorder have higher, perfectionistic, more ambitious goals (Nusslock et al., 2007; Johnson et al., 2012) and goal-striving cognitive style (Francis-Raniere et al., 2006). Moreover, recent research points to significant differences in the drive or how at-risk individuals pursue their goals. For instance, Dempsey et al. (2020) found that the risk of bipolar disorder is associated with higher levels of tenacious pursuit of goals. Both tenacious goal pursuit and positive primary irrational beliefs (e.g., “I must achieve what is important for me”) may be regarded as a form of goal persistence. Thus, it is not surprising that primary irrational beliefs have similar contributions to the risk of psychopathology as tenacious goal pursuit.

Another interpretation of the association between positive irrational beliefs and hypomanic personality is that positive irrational beliefs are cognitive components of hypomanic personality. This interpretation is consistent with the view of primary irrational beliefs as motivational parts of a dysregulated BAS system. Previous research has suggested that primary irrational beliefs (positive demandingness and happiness irrational beliefs) are related to BAS measures (Tiba, 2005; Tiba and Szentagotai, 2005; Yıldırım and Maltby, 2021).Thus, assimilating positive primary irrational beliefs as cognitive components pertaining to the activation of a dysregulated BAS system would explain the association between positive irrational beliefs and the risk of bipolar disorder (Francis-Raniere et al., 2006; Alloy et al., 2009). An oversensitive and dysregulated activity of the BAS has been consistently found in individuals with bipolar disorder (Alloy and Abramson, 2010). Thus, rigidly following goals and desires both in positive and when goals are not met may reflect the activation of BAS. In our study, we did not find a significant relation between the reports of rigidly persisting to attain goals in situations when goals are not met (HABS demandingness) and the risk of hypo/mania. It is possible that irrational beliefs measured by HABS may include persistent avoidance goals that would be related to behavioral inhibition system (BIS) (Tiba, 2005). Yet a visual inspection of the demandingness items from the HABS short form scale do not refer to avoiding aversive situations (e.g., “I should avoid failing at the exam”). Further research should clarify whether rigidly persisting in the pursuit of goals when negative situations block our goals is related or not to the risk of hypo/mania since our results show that general irrational beliefs in negative situations are positively related to the risk of hypo/mania.

Previously, it has been suggested that interventions aiming at reducing tenacious goal pursuit should be added to existing intervention to reduce the risk of bipolar disorder (Nusslock et al., 2009; Dempsey et al., 2020). Our findings suggest that rigid beliefs about attaining goals in response to cues of rewards should also be addressed in order to reduce the risk of developing bipolar disorder. Showing that irrational beliefs are involved in the risk of bipolar disorder brings a major advantage for building interventions for reducing the existing risk for bipolar disorders. There are many studies that consolidate the evidence-base of treatment methods developed for restructuring irrational beliefs (Ellis et al., 2010; David et al., 2018). These methods may be targeted to changing irrational beliefs also in response to positive situations, not only to negative situations. Given that restructuring irrational beliefs may show significant clinical benefits for preventing and reducing depressive episodes (Szentagotai and David, 2010) it may be practical to teach clients to apply similar methods to prevent hypo/manic episodes. However, interventions aiming at changing positive irrational beliefs should undergo empirical clinical tests.

There are several limitations to consider in interpreting the results of the study. The study used validated measures for irrational beliefs and depression. HPS and POG remain to be validated on the Romanian population as the forward and back translations were first made for the present study. Factorial analysis based on current data supported the proposed factorial structure of the scale. We checked for the overlap between measures of HPS and positive irrational beliefs, and found no similar items. Another limitation regards the generalizability of our results to clinical groups. We used a non-clinical sample, with only six participants reporting currently taking medication for affective problems. Similarly, we did not account for family history of depression and bipolar disorder, past depression and other mental illness, or childhood adversity. As these factors could interact with irrational beliefs in determining the risk for bipolar disorder further research should account for them as well. Thus, our conclusions may be interpretable in terms of general population, rather than clinical population. Further research should investigate the role of positive irrational primary beliefs in clinical groups with a diagnosis of bipolar disorder and using longitudinal designs. Similarly, generalizing our results to the male population should be made with caution since 77.6% of our sample was of female participants yet a larger participation of female subjects was expected, since previous studies have typically reported that male sex is associated with non-response to online surveys (Lallukka et al., 2020). Besides these limitations, our results point to primary irrational beliefs in positive situations as a potential route for changing the risk of bipolar disorder and a target for efforts to prevent bipolar disorder.

## Data availability statement

The raw data supporting the conclusions of this article will be made available by the authors, without undue reservation.

## Ethics statement

The studies involving human participants were reviewed and approved by the Ethics Committee of the Faculty of Socio-Humanistic Studies, University of Oradea. The patients/participants provided their written informed consent to participate in this study.

## Author contributions

AT designed the study and wrote the protocol. AT, LV, FB, and DM undertook the statistical analysis and wrote the first draft of the manuscript. AT, MD, ST, CB, FB, DM, LV, SI, and SP revised the manuscript. AT, SP, CB, FB, and DM managed the data collection. All authors listed have made a substantial contribution to the work and approved it for publication.
